# Range extension of *Christisoniascortechinii* from mainland Southeast Asia into Borneo, and notes on the distinction between *Aeginetia* and *Christisonia* (Orobanchaceae)

**DOI:** 10.1186/s40529-015-0109-3

**Published:** 2015-10-05

**Authors:** Antony van der Ent, K. M. Wong

**Affiliations:** 1 Centre for Mined Land Rehabilitation, Sustainable Minerals Institute, The University of Queensland, St. Lucia, QLD 4072 Australia; 2 Laboratoire Sols et Environnement, Université de Lorraine-INRA, UMR 1120, 54000 Nancy, France; 3 Singapore Botanic Gardens, 1 Cluny Road, Singapore, 259569 Singapore

**Keywords:** *Aeginetia*, Borneo, China, *Christisonia*, Kinabalu, Laos, New record, Orobanchaceae, Philippines, Ultramafics

## Abstract

**Background:**

*Christisonia* is a little-documented and poorly studied root-parasitic genus in the Orobanchaceae occurring in India, China, Indochina and part of the Malesian region. Recent collection of a *Christisonia* taxon in Kinabalu Park in Sabah, Borneo, taxonomically identical to earlier Sabah collections that have hitherto not been recorded in the literature, led to an assessment of the taxonomic identity of the species against *Christisonia scortechinii*, *C. siamensis*, *C. sinensis* and related species.

**Results:**

Some taxa in China, Indochina, the Malay Peninsula, and the Philippines are morphologically identical to the Borneo taxon except in the number of calyx lobes, but differ by several distinctive characters from other well-distinguished species in the region. Studies of dried herbarium specimens, augmented by photographic images of different stages of fresh flowering material and a scrutiny of available descriptions confirmed that the calyx has two primary lobes in the bud that may separate into 3–5 portions, giving a variable number of apparent lobes in specimen material collected at different localities. This new scrutiny of the calyx also permitted an improved description of the calyx differences that separate *Christisonia* and the closely related *Aeginetia*, which have not been clearly elucidated in the past.

**Conclusions:**

*Christisonia* *scortechinii* Prain (Orobanchaceae), the only species that was described as having an initially spathaceous calyx among species of this root-parasitic genus, is newly recorded for Borneo (including Kinabalu Park, where its presence has been overlooked). The range of the species in mainland Southeast Asia, previously extended from Peninsular Malaysia to Thailand and Vietnam, is here further extended to Laos and China. *Christisonia wightii* Elmer (relevant to the Philippines) and *C. sinensis* Beck (China) are reduced to synonymy.

## Background

The root-parasitic genus *Christisonia* Gardn. has ca. 20 species distributed from India to China and the Malay Peninsula (Parnell 2001, 2008), Philippines, SW Celebes and Flores (Van Steenis 1967). *Christisonia* has hitherto not been documented for Borneo Island, in spite of pre-existing collections, although the closely related *Aeginetia* L. is recorded there (Beaman and Anderson 2004; under Scrophulariaceae). Both *Christisonia* (Quisumbing 1940) and *Aeginetia* (Kusano 1903, Lee and Goseco 1932) have been reported to parasitize the roots of sugarcane crops. In nature, *Christisonia* is also a root-parasite on other grasses, including bamboos (Barlow 1982; Parnell 2001), and other plant families, including Acanthaceae (Nandikar et al. 2013) and Vitaceae (Benniamin et al. 2012).

**Fig. 1:**
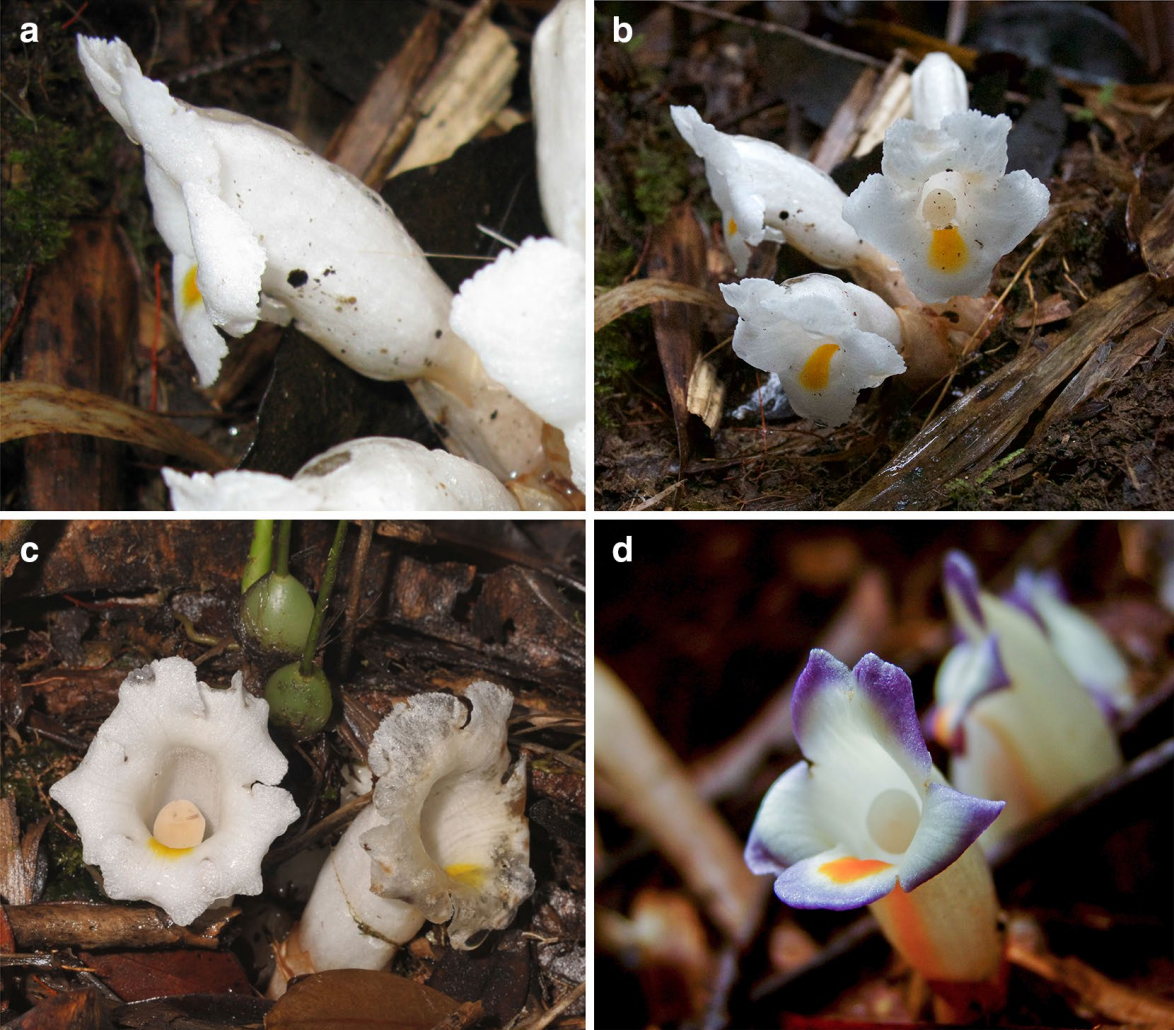
Flowering material of *Christisonia scortechinii* from Sabah (Kinabalu Park) (**a**–**c**) and a *purple*-tinged variant from Peninsular Malaysia (Sungei Wi, Cameron Highlands) (**d**). **a** Side view of flower showing the transparent slime covering. **b** Cluster of fresh flowers with short calyx lobes and a yellow band on the lip that continues down into the corolla tube. **c** Open flowers, with one showing the peltate discoid stigma in the throat. **d** *Christisonia* with unusual *purple*-tinged corolla. Photos: Sukaibin Sumail (**a**), A. van der Ent (**b**, **d**) and R. van Vugt (**c**)

*Christisonia* and *Aeginetia* are resolved in the same clade, within the tribe Buchnereae, in the general molecular phylogeny of the Orobanchaceae (McNeal et al. 2013). However, a closer study of the phylogenetic relationship between these two genera with adequate taxon sampling is still elusive. *Christisonia* species have been distinguished by their distinctly lobed calyx from *Aeginetia*, also a root-parasitic genus, which has spatheate calyx (Parnell 2001). Prain (1904) had, however, also described the calyx of the Malayan *C. scortechinii* as “spathaceous rupturing into usually 2, occasionally 3–4 lobes in the fully opened flower”, so causing uncertainty if the spatheate calyx of *Aeginetia* is a consistent character for distinguishing it from *Christisonia* (Parnell et al. 2014). Nevertheless, the presence of a covering of colourless slime over *Christisonia* flower buds (Fig. 1a) seems to provide another consistent distinction from *Aeginetia*, in which this slime is absent (Parnell et al. 2014). Also, most *Christisonia* spp. have largely white corollas, and most *Aeginetia* spp. lilac, purple, red or yellow corollas, although this is not mutually exclusive and a purplish corolla form has been reported for *C. scortechinii* (Yong 1989) (Fig. 1d) and species such as *A. indica* also have white corolla forms (Parnell 2012). Another general difference is that *Christisonia* flowers are typically on short pedicels borne on short or obscure stems that do not protrude much from the ground level, whereas *Aeginetia* species often have taller stems 10–40 cm long that carry the flowers clearly above ground level.

More recently, the distribution of several *Christisonia* species has become better documented. For example, *C. siamensis* Craib, once thought to be endemic to Thailand (Parnell 2001) has been documented in Nagaland, NE India (Benniamin et al. 2012) and SW China (Tan 2013). Nevertheless, the identification of these root-parasites, the flowers of which are delicate and difficult to preserve (spirit collections are essential), remains challenging. Comparatively few collections of the various species and the poor state of preservation of key reference specimens of earlier documented species hamper comparison and reliable identification (Parnell 2001; Parnell et al. 2014). Only in a number of cases where photographic images of sufficient quantity are available can we elucidate the morphology and variation. This, fortunately, is becoming the case as amateur and professional botanists make their images more readily available on the World Wide Web.

This paper formally records *Christisonia* for Borneo through specimens collected recently from Kinabalu Park by Antony van der Ent (Fig. 1a–c), which began this investigation, as well as previous collections from there and elsewhere in Sabah.

## Methods

Herbarium material of *Christisonia* for Borneo was studied at the Singapore Herbarium (SING) and the Sabah Parks Herbarium (SNP), and through images on JStor for the Global Plants Initiative, the latter including material from K and WU; herbarium acronyms used follow Thiers (2015). The SNP specimens collected by A. van der Ent in Kinabalu Park were preserved in FAA mixture. Photographic images of different stages of fresh flowering material taken by A. van der Ent and colleagues or which were available from various internet sources (as respectively acknowledged below) aided the interpretation of floral structures and development, especially that of the calyx.

## Results and discussion

### *Christisonia* newly recorded for Borneo

Cedric E. Carr, better known for his orchid collecting (Kiew 2003), was apparently the first person to collect a *Christisonia* from Mount Kinabalu and Borneo, although this was never recorded in any publication, including the recently completed *Plants of Mount Kinabalu* where Beaman and Anderson (2004) had missed Carr’s number *SFN 26457* of this taxon in the Singapore Herbarium. Carr’s locality was the Kinataki River (below the famous Marai Parai spur) at around 1400 m on the western flank of Mount Kinabalu.

*Christisonia scortechinii* (Fig. 1) was recorded by Antony van der Ent from two localities inside Kinabalu Park, the first at Marai Parai (near the Kinataki River where also Carr collected this species on Mount Kinabalu over 80 years earlier), and the second on Mount Tambuyukon in the north of Kinabalu Park. Both localities sit at 1500–1800 m asl in dense montane cloud forest on ultramafic soils and have remarkably similar vegetation in aspect and composition. Specimens were collected only from the Marai Parai site on Mount Kinabalu, while the Mount Tambuyukon occurrences were documented with photographs.

The *Christisonia* is root-parasitic on the bamboo *Racemobambus gibbsiae* (Poaceae) which is abundant at both sites. The parasitic plants occur as scattered typically “clumped” individuals in the permanently moist humus-rich soil. It appears to be flowering year-round as it has been recorded on a number of occasions during fieldwork. The Marai Parai site is a steep slope along the Kinataki River below the famed spur that is the type-locality for many of Kinabalu’s enigmatic plant species, such as *Nepenthes rajah* and *N. edwardsiana* (Nepenthaceae). Mount Tambuyukon (2579 m asl) is the third highest mountain on the island of Borneo and the highest ultramafic mountain in the region. The vegetation on this mountain ranges from tall mixed dipterocarp forest on the foot slopes to stunted graminoid scrub near the summit. The *Christisonia* occurs between Musang Camp and General Camp on the east flank roughly 400 m below the summit.

Voucher material for *C.* *scortechinii* recorded on Mount Kinabalu include the following:

SABAH, Ranau, Mount Kinabalu, Kinataki River, ca. 4500 ft (1370 m), 7 Mar. 1933, *C.E. Carr SFN 26457* (SING), Marai Parai, near Kinataki River, 1630 m, 31 Mar. 2011, *A.* *van der Ent, S. Sumail & R.M. Karim SNP 23658* (SNP).

*Christisonia scortechinii* is also known from elsewhere in Sabah, also on ultramafic soils:

SABAH, Kinabatangan district. Telupid, Bukit Tawai, near summit, 1 Sep. 1999, *R. Kiew RK 4817* (SING), Sungai Ruku-Ruku Forest Reserve, 3 Sep. 1999, *Ali Ibrahim & R. Kiew AI 540* (SING).

The flora of Kinabalu Park is the richest globally in terms of the number of plant species per unit area with more than 5500 taxa recorded from an area roughly 1200 km^2^ in extent (Beaman 2005, Van der Ent unpublished). Plants with alternative metabolism or nutrient-acquisition strategies in Kinabalu Park include insect and/or rodent scat trapping (‘carnivorous’) plants (Nepenthaceae, Droseraceae, Lentibulariaceae), myrmecophytes (Rubiaceae: *Hydnophytum*, *Myrmecodia*), myco-heterotrophic (‘saprophytic’) plants (mainly Burmanniaceae, Orchidaceae, Triuridaceae), and parasitic plants (principally Loranthaceae and Rafflesiaceae). Most of the hemi-parasitic plants (those that have an active photosynthesis system) in Kinabalu Park are in the order Santalales represented by approximately 60 species predominantly in the families Loranthaceae and Santalaceae (Beaman and Anderson 2004). Apart from the newly recorded *Christisonia*, holo-parasitic plants (which derive all carbohydrates exclusively from the host) include three species of *Balanophora* (*B. lowii, B. reflexa* and *B. papuana*) (Balanophoraceae), one species of *Rhizanthes* (*R. lowii*), one species of *Mitrastema* (*M. yamamotoi*) and two species of *Rafflesia* (*R. keithii* and *R. pricei*) (the latter three genera in Rafflesiaceae).

The local occurrence of ultramafic soils has been suggested as a driving force for speciation, in combination with recent uprising of the Mount Kinabalu Massif, isolation and other factors (Beaman and Beaman 1990). Soils developed from ultramafic (often called ‘serpentine’ by botanists and ecologists) bedrock induce an edaphic filter on the vegetation that includes nutrient deficiencies, major cation imbalances and metal toxicities (Proctor 2003). As a consequence, ultramafic vegetation in Sabah is often of lower stature, with distinct species composition compared to mixed dipterocarp forest on sedimentary soils. Ultramafic habitats in Borneo are known to harbour a multitude of rare and localised plant species (Wong 1998). Although ultramafic host a number of endemic taxonomic entities that could be evolutionarily derived from closely related taxa from adjacent environments, so-called “neo-endemics” (Van der Ent et al. 2014, 2015), we could not detect any consistent morphological differences between the *C. scortechinii* from the Sabah ultramafic sites and that from elsewhere.

### Comparing Sabah *Christisonia* material with closely related taxa

At the outset we were able to determine that the Sabah taxon (Fig. 1a–c) is most related to *C. scortechinii* Prain, *C. siamensis* Craib and *C. sinensis* Beck, in that all these typically or frequently have glabrous white corollas with a single yellow patch that runs from the base of the lip to within the throat and peltate stigmas.

### *Christisonia scortechinii*

An unnumbered collection of *C. scortechinii* at Kew (K000899781) represents potential type material against which to interpret the species in its original Malay Peninsula provenance, given the type sheet bearing Scortechini’s number 2121 at Calcutta is in poor condition (Parnell et al. 2014). But even on the Kew sheet, it is difficult to make out intact calyx lobes and verify other characters because the dried material is in poor condition and mounted on a sheet. Thus the interpretation of *C.* *scortechinii* has depended in large part on direct visual assessment of fresh material from Peninsular Malaysia (Fig. 1d), other herbarium material available (see below), the original description by Prain (1904), as well as the sketch in Henderson (1954) and available photographic images, such as in http://www.parasiticplants.siu.edu/Orobanchaceae/images/Christisonia1.jpg and http://www.parasiticplants.siu.edu/Orobanchaceae/images/Christisonia3.jpg.

### *Christisonia siamensis*

*Christisonia siamensis* is well distinguished from *C. scortechinii*, *C. sinensis* and the Sabah taxon in frequently having violet-purplish corolla coloration and a distinct hair tuft at the insertion of the anther that obstructs the corolla throat (Parnell et al. 2014), which is absent in the latter three taxa, which have quite glabrous filaments.

### *Christisonia sinensis*

**Fig. 2:**
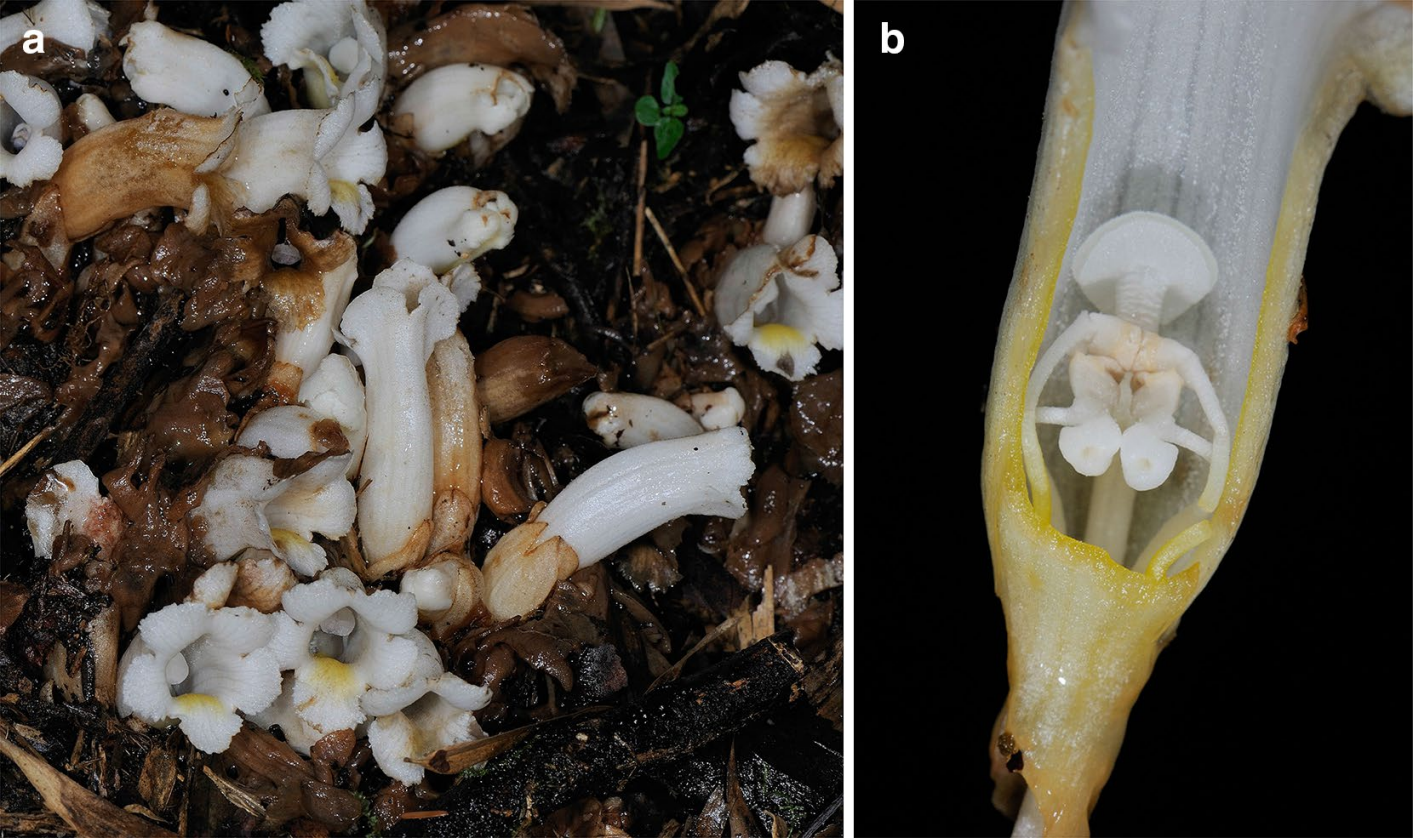
*Christisonia sinensis* from Chinese material. **a** Flowers, with slime over young buds and (*center-right*) suturing of calyx lobes that incompletely separate. **b** Corolla cut open to show the peltate discoid stigma and two pairs of stamens; in the shorter stamens, the anther appendage has a short acute projection on the dorsal side. (Reproduced with the kind permission of Ming-I Weng, from: https://www.flickr.com/photos/mingiweng/4927639081/; http://www.flickriver.com/photos/mingiweng/4928219216/)

*Christisonia sinensis*, found in SW and SE China, was reduced to the synonymy of *C. hookeri* C.B.Clarke ex Hook. in the Flora of China (Zhang and Tzvelev 1998). However this may not be justified because, as pointed out by Parnell et al. (2014), the stigma was described as unequally bilobed in the type description of *C. hookeri* (Hooker 1885) whereas the Flora of China treatment describes a discoid stigma 4–6 mm in diameter, which fits the peltate stigma illustrated in photographic images from Chinese material, as shown in Fig. 2, for example.

Although Parnell et al. (2014) expressed doubt that Chinese material (with peltate stigmas) had been correctly identified as *C. hookeri* (described as having bilobed stigmas), they did not comment on the status of the name *C. sinensis*.

### Identification of the Sabah *Christisonia*

Table 1 compares a number of characters among the Sabah taxon, *C. scortechinii*, *C. siamensis* and *C. sinensis*, as well as *C. calcarata* and *C. hookeri*, both of which are clearly differentiated from the others by their bilobed stigmas. Other very conspicuous and consistent characters also help differentiate consistently recognised species in Table 1, such as the slender pedicellate flowers of *C. calcarata*; a distinct hair tuft at the anther insertion in *C. siamensis*; and the densely pubescent corolla in *C. calcarata*. It would appear that while the predominantly white corolla is also important, occasional violet or purplish tinges do occur. The single band of yellow marking the lip and down into the corolla throat in *C. scortechinii* (Fig. 1), *C. siamensis* and *C. sinensis* (Fig. 2a) also distinguishes these species from *C. calcarata*, where the entire throat is yellow. These provide an idea of various specialised characters that clearly distinguish species.

**Table 1:** The Sabah *Christisonia* compared with five other taxa

	Sabah *Christisonia*	*C. scortechinii*	*C. siamensis*	*C. sinensis*	*C. calcarata*	*C. hookeri*
Pedicel	Short-thick, ca. 1 cm long	Short-thick, ca. 1 cm long	Short-thick, ca. 1 cm long	Short-thick, ca. 1 cm long	*Slender, 2*–*3.5* *cm long*	Short-thick, ca. 1 cm long
Calyx colour	White	White to purplish	White, yellowish to dark purple	White	Pale yellow	Violet
Calyx surface	Glabrous	Glabrous	Glabrous	Glabrous	Glabrous	Glabrous^a^
Calyx lobes/teeth	2–4 (–5)	2–4 (–5)	(2–) 3 (–4)	(2–) 3–4 (–5)	5	(4–) 5
Enclosure of corolla tube by calyx (herbarium material)	About half	Less than half	Less than half	About half	Less than half, to half	About half
Enclosure of corolla tube by calyx (fresh material)	About a quarter	A quarter to a third	A third to half	A quarter to a third	A quarter to a third	?
Corolla colour	White	White to tinged with violet	White tinged with violet	White	White	White with violet seams
Corolla pubescence	Glabrous	Glabrous	Glabrous	Glabrous	*Tube outer surface and both sides of lobes white*-*pubescent*	Glabrous
Corolla lobe & throat colour	White, with yellow-orange patch from base of lip down to inside the tube	White, with dark yellow patch from base of lip down to inside the tube	Bluish to violet, with yellow patch from base of lip down to inside the tube	White to slightly lilac, with yellow-orange patch from base of lip down to inside the tube	White to greyish or bluish, *bright yellow patch on all lobes to down inside the tube*	?
Hairs at point of attachment of anthers	Glabrous	Glabrous	*Distinct hair tuft* obstructing corolla throat	Glabrous	*Sparse scattered short hairs on filaments*	Glabrous
Anther appendage, dorsal side	With short acute projection	With “projecting acute posterior process” (Prain 1904)	With short acute projection	With short acute projection	*With a slender spur*-*like projection*	*Rounded, without acute projection*
Stigma	Peltate	Peltate	Peltate	Peltate	*Bilobed*	*Bilobed*
Known distribution	N Borneo (Sabah)	Peninsular Malaysia, Thailand, Vietnam^b^	Thailand (N NE, SW), India (Nagaland)	SW and SE China	India, Pakistan	India
Habitat	Montane sites over 2000 m	Montane sites ca. 650–1700 m	Mixed deciduous forest at 300–800 m	Montane forest, 1500–2000 m	Montane sites	Montane sites

^a^Not pubescent as stated in Benniamin et al. (2012)

^b^Material from Thailand (N, NE, SE, Peninsular, from ca. 1200–1655 m) and a more northerly latitude in C Vietnam (642 m) placed in *C. scortechinii* by Parnell et al. (2014)

It can be seen from Table 1 that, while *C. calcarata*, *C. hookeri* and *C. siamensis* are immediately distinguished from the rest as stated above, *C. scortechinii*, *C. sinensis* and the Sabah taxon remain rather similar, including in the variable number of calyx lobes, a character that had caused much hesitation in deciding whether or not *C. scortechinii* from the type provenance (Peninsular Malaysia) and other provenances in Thailand and Vietnam should be conspecific.

In order to understand the nature of the calyx lobes better, we examined specimen material of *C. scortechinii* from Peninsular Malaysia (the type provenance) to see if they agreed with the original description of Prain (1904); and both dried preserved material (mostly expected to be in poor condition) and photographic images that could assist in interpreting this character for other similar taxa in China (as *C. sinensis* Beck or *C. hookeri* sensu Zhang & Tzvelev, non (C.B.Clarke) Hook.), Vietnam, and Thailand.

For material from the type provenance of *C. scortechinii*, the diagram given by Henderson (Henderson 1954: 335, Fig. 317) clearly shows calyces with 2 or 3 lobes. Earlier, Ridley (1923) had also described “Calyx spathaceous splitting into 2–4 lobes…”, which is not different from what Prain (1904) stated. We also studied the following material:

PENINSULAR MALAYSIA, Perak, Bukit Kinta Forest Reserve, 28 Apr. 1987, *R. Kiew RK 2583* (KEP, SING); Perak, Ulu Batang Padang, no date, *Ridley 13693* (SING); Selangor, Ginting Simpah, top of pass, ca. 2000 ft (609 m), 20 Sep. 1947, *J. Reid s.n.* (SING).

In fact, Reid’s specimen had helped the description in Henderson (1954), as evident from his letter to Henderson pasted on the specimen, which, among other things, gave the occurrence of the species as “sparsely distributed in small localised patches at moderate altitudes.” All five calyces of young as well as open flowers on the Reid specimen sheet show 2 lobes; one young bud shows a single deep suturing across the top of the calyx (implying two lobes from a younger stage). The Ridley specimen has only one ‘good’ flower in the packet showing clearly two main lobes, each with a short median tear (which can be expected to give at least four lobes perceptible at a later stage). The Kiew specimen shows 2, 3, 4, or 5 calyx lobes, unequal in width as if from tears rather than from preformed equivalent calyx members.

*Christisonia sinensis* is very similar to *C. scortechinii*, including some Thai and Vietnamese (Central Mountains Region) material that was identified as the latter species (Parnell et al. 2014). The wider concept of *C. scortechinii* (Parnell et al. 2014) accepted that the calyx may be spatheate in the bud but becoming 2–4(–5)-lobed. Although herbarium material for the genus is admittedly poor (Parnell et al. 2014), the type of *C. sinensis*, *Handel*-*Mazzetti 9595*, Yunnan, 2850 m, 31 July 1916 (WU), clearly shows the short thick pedicels, short-lobed calyces and peltate stigmas that accord with the images of material from China as well as the Thai and Vietnam material mentioned. Moreover, the type also includes flowers where the calyx is variably 2–3-lobed or even 5-lobed, indicating that the calyx in *C. sinensis* does not always part into 5 lobes.

**Fig. 3:**
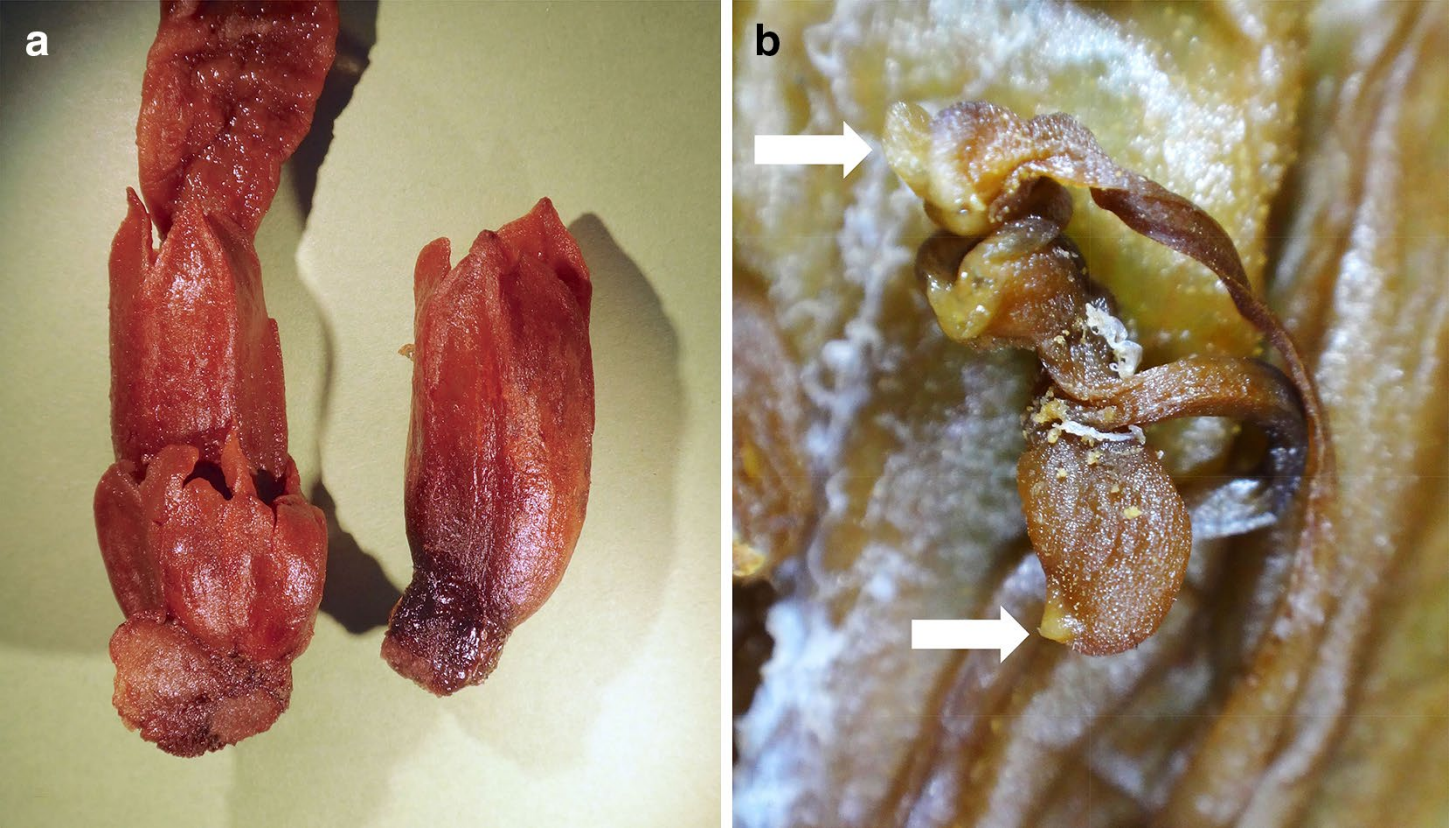
*Christisonia scortechinii* from Kinabalu Park, showing calyces separating into 3 or more lobes (**a**) and detail of one set of stamens (**b**) showing anther of longer stamen (*upper arrow*) close to the anther of shorter stamen, which has a dorsal lobe with an acute process at its tip (*lower arrow*). From spirit-preserved material of *Van der Ent* et al*. SNP 23658* (SNP)

Prain (1904) described that the lower pair of stamens of *C. scortechinii* had “a projecting acute posterior process”, and *C. sinensis* material from China, as well as Thai and Vietnam material ascribed to *C. scortechinii* in Parnell et al. (2014), also develop a small acute or conical projection on the anther appendage. This can be seen in Chinese material (Fig. 2b) as well as the Sabah material (Fig. 3b). These vary slightly in prominence but are instantly distinguished from the slender elongate, spur-like projection from the dorsal side of the anther appendage developing in another species, *C. calcarata*.

Fresh Chinese material from Taiwan referable to *C. sinensis* beginning with two calyx lobes that sustain visible apical tears can be seen in images from the website: https://www.flickr.com/photos/mingiweng/4928220164/ and examples of calyx tearing into 3–5 lobes can be seen in: http://www.plant.csdb.cn/details?guid=photo:cfh@5c7f74b1-31df-4c72-a872-a2d446a8fa22.

Incompletely separating calyx lobes are clearly seen in Fig. 2a.

Even more instructive, Chinese (Taiwan) material showing a bud calyx with only a small apical opening, and other calyces tearing irregularly into several lobes are obvious in: http://blog.xuite.net/bibibear599/twblog/137870776.

Likewise, the variable number of calyx lobes that can be seen in web-images of fresh material from mainland China, Vietnam and Thailand has been discussed in detail by Parnell et al. (2014), including their Figs. 1c and 2a. Vietnam material with calyx tearing into 5 or more lobes is also available in: https://www.flickr.com/photos/biodivn/14351717859/.

To these we wish only to add a new record of the same taxon for Laos:

LAOS, Houa Phan Province, Vieng Thong District, Nam Et-Phou Louey Natural Protected Area, Nam Et side, near Ban Phon Song, 20°20′46″N, 103°22′04″E, 953 m, 8 Jun. 2013, *J. Leong*-*Skornickova, M. Rodda, Aung Thame, U. Souvannakhoummane, K. Phoutthavong, M. Norsaengsri & S. Sangvirotjanapat JLS 2407* (E, P, QBG, SING).

Most calyces from this specimen are torn, but one clearly shows 2 lobes, another in bud has the calyx showing only a single small median tear (or slit).

These observations imply that there is no consistent difference in the number of calyx lobes between *C. scortechinii* and *C. sinensis*, supporting a key premise on which Parnell et al. (2014) based their extension of the range of *C. scortechinii* to include sites in Thailand and Vietnam. Although for some other species, e.g., *C. calcarata*, the number of calyx lobes seems evenly formed and are consistently 5 in number, for other species such as *C. scortechinii* and *C. sinensis*, there is generally a variable number of calyx lobes. It would seem that the basic condition representative of the latter two taxa is a basically 2-lobed calyx, where the lobes are fused to only slightly separated in the bud stage, later separating into two clear lobes, each of which can sustain a secondary median tear, in all giving 2, 3, or 4 lobes (as was recorded by Prain for typical *C. scortechinii* and not authoritatively recorded for *C. sinensis*), or even 5 lobes (as common in and recorded for *C. sinensis* and some material from Vietnam and Thailand). Other distinct species may also show a similar calyx character, e.g., the painting of *Christisonia thwaitesii* Trimen by H. DeAlwis in Trimen (1893–1898), t. 69, depicts calyces with 2 lobes, 3 lobes and irregularly tearing lobes: http://plantillustrations.org/illustration.php?id_illustration=157328&mobile=0.

In fact, the description of the calyx by Trimen (1895: 263) of *C. thwaitesii* is not only accurate but also describes the condition in *C. scortechinii*: “…quite closed in the bud (which is beaked), deeply 2-lipped, the lips irregularly or faintly 2- and 3-toothed…”.

The material from Sabah also amply displays the same condition of basically two calyx lobes that may further divide (tear) to give 2, 3, or 4, even 5 lobes. Carr’s material is rather well preserved, with one calyx clearly showing division into two main lobes, and another having two main lobes with apical tears. The recent material collected by van der Ent and colleagues also shows 3–4 calyx lobes (Fig. 3a).

### A wider geographical range for *Christisonia scortechinii*

The impression from descriptions, dried herbarium specimens and photographic images of different stages of fresh flowering material in *C. scortechinii*, *C. sinensis* and the Sabah taxon is that the calyx is basically composed of two lobes that slightly to mostly cohere in the young flower bud, with an apical slit along which the main calyx lobes part and through which the corolla protrudes. In summary, there seem to be no significant differences between *C. scortechinii* and *C. sinensis* and our Sabah taxon. The Malayan name would take precedence.

*Christisonia scortechinii* is probably also distributed in the Philippines. Images by Leonardo L. Co identified as *C. wightii* Elmer from the Sierra Madre Mountain Range, Cagayan Province, Luzon Island, and the (ultramafic) Mount Victoria massif on Palawan Island (http://www.philippineplants.org/Families/Orobanchaceae.html), both show *Christisonia* flowers with a peltate discoid stigma, and appear to be the same as *C. scortechinii*. *Christisonia wightii* Elmer, described based on material from Negros, was the only species described for the Philippines, and of which the type is not found and probably not extant (likely at PNH and destroyed during WWII). Of the type specimen *Elmer 9510*, Elmer (1915) says “A specimen was however sent…to *Dr. Ridley* [italics his] for determination and who pronounced it a species of *Christisonia*.” This collection is not at SING. We have examined the following material, however most calyces are fragmented:

PHILIPPINES, Mindanao, Camp Keelthler, Lake Loamao, Apr. 1907, *M.S. Clemens, s.n.* (SING).

Elmer (1915) described the stigma as “composed of 2 broad and fleshy lobes”, which differs from the clearly peltate discoid stigma in *C. scortechinii*, otherwise the details he gives do not vary specially from the characters of *C. scortechinii* or *C. sinensis*. We regard that Elmer’s description of the stigma may have been inaccurate and consider that his species is likely the same as *C. scortechinii*.

The material from China, Vietnam and Thailand (as detailed by Parnell et al. 2014), Laos, Peninsular Malaysia (Prain 1904; Ridley 1923) and Borneo (Sabah), probably also that from the Philippines which we identify as *C. scortechinii* all share the following characters: short thick flower pedicels barely 1 cm long; a calyx with 2 primary lobes in the bud that may separate into 3–5 lobes; a typically white, glabrous corolla with a distinctive yellow–orange patch on the lip that continues down into the corolla tube; also additionally, an anther appendage with a short acute-conical projection on the dorsal side (Figs. 2b, 3b). Furthermore, as with the Peninsular Malaysia, Sabah and Philippine records, the ecological distribution of the taxa here included into *C. scortechinii* is clearly montane: the Thai records were from 1200 to 1655 m, the Laos record was from 953 m, the Vietnam record from 642 m at Bi Doup in the Central Mountains Region (Parnell et al. 2014), and the Chinese account generally recorded 1500–2000 m for the species (as ‘*C. hookeri*’) (Zhang and Tzvelev 1998).

### A corollary by way of conclusion: *Christisonia* vs *Aeginetia*

Our current understanding of how the calyx structure varies within *C. scortechinii* allows us to recognise it as a species with the main part of its range from the Malay Peninsula to parts of Thailand, Vietnam, Laos and south China, and which is now recorded for Borneo (Sabah) and probably found also in the Philippines. We thus interpret the species to be vicariant across the South China Sea, between mainland Southeast Asia and the Philippine islands and Borneo. Correspondingly, *C. sinensis* and *C. wightii* should be regarded as synonyms of *S. scortechinii*:

*Christisonia scortechinii* Prain (1904: 205); Ridley (1923: 489); Henderson (1954: 335); Parnell et al. (2014: 16–23). Type: MALAY PENINSULA, Perak, no date, *Scortechini 2121* (holotype CAL, barcode 0000025048; possible isotype K, barcode K000899781).

Synonyms:

*Christisonia* *wightii* Elmer (1915: 2793). Type: PHILIPPINES, Negros, Oriental, Dumaguete (Cuernos Mountains), Mar. 1908, *Elmer 9510* (holotype PNH, not found, probably destroyed).

*Christisonia* *sinensis* Beck (1930: 314). Type: CHINA, Yunnan, 2850 m, 31 Jul. 1916, *Handel*-*Mazzetti 9595* (holotype WU, barcode WU0060576).

*Christisonia hookeri* sensu Zhang and Tzvelev (1998: 242), non Hooker (1885).

It was perhaps unfortunate that Prain (1904) had chosen the term “spathaceous” to describe the calyx of *C. scortechinii*. Use of this term has also led to some concern that an initially “spathaceous” calyx in *C. scortechinii*, the sole example of this in a genus where generally the species were described as having several (up to 5) discrete calyx lobes, would weaken the distinction between *Christisonia* and *Aeginetia* by their otherwise different calyx forms.

In fact, in *C. scortechinii* the two primary calyx lobes (which may remain intact or further tear to give more lobes) are subequal and so ensheath the emerging corolla tube at the beginning; this character is therefore rather different from the conspicuously spathaceous calyx in *Aeginetia*. In *Aeginetia*, there is only one (incomplete) split developing along the ventral median in the conspicuously curved calyx, through which the corolla emerges. Thus, the genera *Aeginetia* and *Christisonia* are still readily distinguished from their calyx form and the way it ruptures during floral development. We venture a distinction between these two otherwise very closely related genera as follows:

- Flower buds covered in a translucent jelly-like slime; flowers typically on short pedicels borne on short or obscure stems that do not protrude much above ground; calyx not or only slightly curved, with 2-several distinct primary lobes, if only 2 then the lobes subequal but possibly dividing further through apical tears; corollas largely white outside, sometimes infused with pink or blue-violet to various degrees ….. *Christisonia*
- Flower buds without a slimy covering; flowers carried on taller stems typically 10–40 cm above ground level; calyx markedly curved or unequally distended, developing only a single split along the ventral median and therefore effectively without distinct lobes; corollas lilac, purple, pink, red or yellow, less frequently white ….. *Aeginetia*
